# Early discharge for patients with benign pleural effusions using a Wayne catheter (pigtail) chest drain - analysis of safety, complications, and quality of life

**DOI:** 10.1590/0100-6991e-20213139

**Published:** 2022-02-18

**Authors:** ANDRÉ MIOTTO, PEDRO AUGUSTO ANTUNES HONDA, DANIELA CRISTINA ALMEIDA DIAS, JORGE HENRIQUE RIVABEN, MARCIO BOTTER, BRUNO FERNANDO BINOTTO, JULIO MOTT ANCONA LOPEZ

**Affiliations:** 1 - Instituto Prevent Sênior, Serviço de Cirurgia Torácica - São Paulo - SP - Brasil

**Keywords:** Pleural Effusion, Outpatients, Thoracic Surgery, Derrame Pleural, Cirurgia Torácica, Pacientes Ambulatoriais

## Abstract

**Objective:**

to assess safety, efficacy and quality of life in patients with benign pleural effusions undergong pleural drainage with Wayne pleural catheter (DW) in an outpatient setting.

**Method:**

this is a prospective study, in which 47 patients were evaluated between July 2017 and October 2018. Patients with non-malignant pleural effusions underwent pleural drainage with clinical evolution compatible with outpatient care were included. Patients who underwent drainage due to other conditions and patients were excluded.

**Results:**

*a*fter catheter placement, the mean length of hospital stay was 3.14 (± 3.85) days, and 21 patients (44.68%) were discharged within 24 hours. The mean time with the catheter was 12.63 (± 7.37) days. The analysis of the pleural fluid was transudate in 87.3% of cases and exudate in 12.3%. The causes of pleural effusion were heart failure (72.3%), renal failure (19.1%), liver failure (6.3%) and pneumonia (8.5%). The quality of life, analyzed according to the parameters of the questionnaire SF 36, showed low average values when compared to other studies. Analyzing each descriptor, the average was greater only in the limitation related to physical aspects. In the other descriptors, the results were similar, but smaller*.*

**Conclusion:**

the outpatient use of pleural catheters of the Wayne type (pigtail) proved to be feasible, safe and with a low associated infection rate. This is a viable option for selected patients.

## RESUMO

**Objetivo:**

avaliar a segurança, a eficácia, as complicações e a qualidade de vida da alta precoce e acompanhamento ambulatorial de pacientes com derrames pleurais benignos submetidos à drenagem pleural com dreno de Wayne (DW).

**Método:**

estudo prospectivo, em que foram avaliados 47 pacientes entre julho de 2017 e outubro de 2018. Foram incluídos os pacientes com derrames pleurais não malignos, submetidos a drenagem pleural com evolução clínica compatível com o cuidado ambulatorial. Foram excluídos os pacientes submetidos a drenagem por outras afecções.

**Resultados:**

após a drenagem, a média do tempo de internação dos pacientes foi de 3,14 (±3,85) dias, sendo que 21 pacientes (44,68%) tiveram alta em até 24 horas após a drenagem. O tempo médio de permanência com o dreno foi de 12,63 (±7,37) dias. A análise do líquido pleural mostrou tratar-se de transudato em 87,3% dos casos e de exsudato em 12,3%. Dentre as causas do derrame pleural, destacaram-se insuficiência cardíaca (72,3%), insuficiência renal (19,1%), hepatopatias (6,3%) e pneumonias (8,5%). A qualidade de vida, analisada segundo os parâmetros do questionário SF 36, teve valores médios baixos, principalmente em relação a outros estudos. Na análise de cada descritor, a média apresentou-se maior somente na limitação por aspectos físicos. Nos demais descritores, os resultados foram semelhantes, mas menores.

**Conclusão:**

o uso ambulatorial de cateteres pleurais do tipo Wayne (pigtail) mostrou-se factível, seguro e com baixa taxa de infecções associadas. Trata-se de opção viável para pacientes selecionados.

## INTRODUCTION

Pleural effusions are frequent causes of dyspnea, with an approximate number of 1.5 million new cases per year. Management can be performed in different ways, depending on the nature and etiology^1^. In the last decade, many studies have been published on the care of patients with neoplastic pleural effusion, but few studies have focused on benign effusions, whose care is still based on classic publications or with a low level of evidence^2^. As a result, most of the evidence found for the care of malignant pleural effusions is applied to patients with benign ones, also due to the lack of specific guidelines^2^. 

Among the most common procedures in patients with pleural effusion, thoracentesis and pleural drainage stand out^1^. Tubular pleural drainage has been used for a long time. Although Hippocrates was the first to report and treat a pleural empyema in 400 BC, there is evidence for the treatment of this condition as far back as 3000 BC, in Egypt. Pleural drains are also used to drain pneumothorax and pleural effusions, with proven efficacy^1^ ^-^ ^3^. The study and advancement of technology has allowed us to reach increasingly effective and safer treatments. A modernization of the classic tubular drain is the Wayne catheter (WC), popularly known as pigtail catheter, due to the curled tip shape. Using smaller gauges than those normally used in tubular drains, the WC, when adapted to the Heimlich valve, allows patients undergoing pleural drainage to be treated well outside the hospital setting, according to the British Thoracic Society Pneumothorax Management Guide^4^. It was initially developed for pneumothorax drainage, but it is widely used for the drainage of pleural effusions, because of its easy handling and since it causes less pain compared with the tubular drain^4^ ^,^ ^5^.

An adaptation of the WC was developed about 10 years ago for prolonged use, with characteristics similar to long-term vascular catheters. These catheters are implanted with the Seldinger technique, followed by subcutaneous tunneling. They are cited in the literature as indwelling pleural catheters (IPC). IPC are an important therapeutic option to treat neoplastic pleural effusions, especially in patients with pulmonary entrapment. They have been evaluated in large, randomized studies and systematic reviews, and have been considered cost-effective in recent works. The main feature is the possibility of home use^6^ ^-^ ^9^. 

The use of IPC in pleural effusions caused by advanced heart disease, liver disease, and renal failure has also been the subject of a recent systematic review, which suggests their use as a viable and effective measure^10^ ^,^ ^11^. Such evidence leads us to believe that patients can remain with pleural drains outside the hospital environment. There is also evidence that pleural drainage in patients with chronic diseases is safe and brings respiratory comfort more quickly^12^ ^-^ ^14^. There remains the question about the safety and effectiveness of the WC’s outpatient use. Evidence on this subject is scarce, and it involves other diseases besides benign pleural effusions^15^. The aim of this study is to evaluate the safety, efficacy, and quality of life in patients with benign pleural effusions undergoing pleural drainage with WC in an outpatient setting.

## METHOD

We conducted a prospective, observational study, evaluating 47 consecutive patients between July 2017 and October 2018. The patients were evaluated at the thoracic surgery outpatient clinic of Hospital Sancta Maggiore - São Paulo, SP - Brazil, by the same researcher. This work was approved by the Ethics in Research Committee - Instituto Prevent Senior (IPS) under number 2,095,297. 

We included patients undergoing pleural drainage for benign pleural effusion. Patients agreed to participate in the research by signing an informed consent form. 

We excluded patients submitted to drainage for other conditions (pleural or multimetastatic neoplasms, pneumothorax, etc.), patients with a low level of consciousness or disease that prevented them from understanding and accepting the research (even with the help of a family member or caregiver) and patients with a history of cancer (active or past).

Patients were diagnosed with pleural effusion by chest radiography and chest computed tomography, confirming clinical suspicion. In the first episode of pleural effusion, patients underwent thoracentesis. Drainage with WC was performed in patients with recurrent pleural effusion who had previously undergone thoracentesis or had large primary pleural effusions with acute respiratory failure. After each procedure, pleural fluid was sent for biochemical analysis, culture, cytology, and patients underwent control chest radiography. Benign pleural effusions were defined as those in individuals without a history of neoplasia, without signs suggestive of neoplasia on clinical and tomographic examination, and transudative pleural effusion or uncomplicated neutrophilic exudative confirmed by laboratory analysis, according to Light’s criteria^1^, in addition to absence of neoplastic cells in pleural fluid cytology. 

All patients underwent pleural drainage with a COOK® 14 French WC, in a hospital environment under aseptic technique and local anesthesia with 2% lidocaine. Catheters were connected to a sterile, closed-system collection bag for better quantification and care. After clinical and baseline improvement, and only after adequate lung expansion seen on chest X-ray, individuals were discharged from the hospital, with an outpatient return scheduled within 10 days. Upon discharge, we oriented all about care with the drain, quantification of drain output, and daily dressings. The guidelines were reinforced by a printed form and delivered to the companion at the time of discharge. Drain output was not a limiting factor for hospital discharge. 

At outpatient return, we evaluated patients and removed the catheter when the output was less than 150mL in 24 hours. In cases of high output, patients were reassessed weekly. After removal, individuals answered a questionnaire on quality of life. After this consultation, patients were followed up via electronic medical records to assess drainage related complications within 180 days. 

We consolidated the obtained data and submitted it to statistical analysis, with calculation of the percentage of events in analytical variables and means and standard deviation in numerical ones. We performed sample calculation based on the population covered by the health care provider, but as this is a pragmatic study, we evaluated all individuals who met the inclusion criteria in the chosen period^18^. In addition to the analysis of epidemiological data, we assessed clinical data that included the cause and nature of the pleural effusion, volume of drained fluid, and complications such as pain, infection, and need for readmission after drain removal. We compared patients by sex and assessed the influence of smoking on the analyzed variables. To compare the results, we adopted the chi-square test, with a significance level of 5%. The chi-square was statistically significant with p<0.001, indicating an association between these categories. To verify whether smoking influences length of stay, drainage time, and complications, we applied the non-parametric Kruskal-Wallis test. 

We chose to assess pain through the use of analgesics during the period with the catheter, as this represents a longer follow-up period than the assessment only at the time of medical consultation. Thus, we divided patients into 3 groups as for the use of analgesics: frequent use (according to the prescribed time), occasional use (only when in pain) and those who did not use analgesics. All were equally instructed to take analgesics in case of pain.

We performed quality of life analysis using the SF 36 questionnaire, widely used in research on quality of life and previously used in patients with pleural effusion^16^ ^,^ ^17^. Patients answered the questionnaire between three and 34 days after WC placement, in the outpatient reassessment after discharge.

## RESULTS

We included 47 patients, 22 men (46.8%) and 25 women (53.2%), with a mean age of 77.27 years (range 57-95). The most common causes of pleural effusion were heart failure (72.3%), renal failure (19.1%), liver diseases (6.3%), and pneumonia (8.5%). All patients were being treated for some non-neoplastic comorbidity. 

After drainage, the mean length of hospital stay was 3.14 (±3.85) days, with 21 patients (44.68%) being discharged within 24 hours after drainage. Ten patients (21.27%) required readmission within 180 days after drainage, eight of these (80%) due to causes unrelated to pleural effusion.

The mean initial volume drained was 1,163.82 (±551.35) milliliters and the mean time spent with the catheter was 12.63 (±7.37) days. The analysis of the pleural fluid showed transudate in 87.3% of the cases and exudate in 12.3%. Pleurodesis was not performed in any of the cases. Most patients had chronic systemic diseases as the cause of pleural effusion, such as heart failure or renal failure, conditions of high prevalence in individuals in the studied age group. The high mean volume of the initial drainage of 1,163.82 milliliters is compatible with all patients showing improvement in their respiratory and general conditions after drainage. Due to the good evolution of the patients, the benign nature of the pleural effusions and the potential risks involved, we chose not to perform pleurodesis. Only two patients were readmitted after drain removal due to recurrence or complications related to pleural effusion. Accidental catheter removal occurred in 2 cases, without serious complications. 

As for complications, there were two cases (4.25%) of skin infections that required treatment with oral antibiotic therapy. One of these patients had poor self-care and hygiene, which possibly contributed to the condition. There were no cases of pneumonia or pleural infections. Only one patient had a positive pleural fluid culture (Staphylococcus aureus), which was not compatible with the clinical picture, as this was a patient who did not present clinical signs of infection and was not using antibiotics. We attribute this result to sample contamination. Regarding pain, 65.95% of patients used analgesics occasionally, 10.65% used them frequently, and 23.40% did not use analgesics.

Patients were followed by the team until the removal of the pleural catheter, and we evaluated long-term outcomes up to 180 days after drainage. During this period, 40.4% of patients died, but none from a cause related to the procedure. Another complication observed was the obstruction of the catheter by fibrin clots, which led 13.63% of patients to seek the emergency room. This situation was easily corrected with aspiration or washing with sterile physiological saline solution. Emergency room physicians were previously instructed on the use and maintenance of catheters and consulted the thoracic surgeon on duty in cases of complications or doubts. No patient required catheter replacement. 

Table 1 shows the demographic data, as well as complications. There was no significant difference between men and women, except for the frequent use of analgesics, more common in women. Table 2 shows complications, length of stay, characteristics, and drainage volume according to smoking. There was no significant difference between groups in all variables analyzed.

**Table 1:** Comparison of demographic data, drain use and complications between men and women who underwent prolonged pleural drainage for benign pleural effusion who were discharged with the drain. Variables were compared using the chi-square test, adopting a significance level of 5%.

	Men	Women	Total	p
Data	n=22 46.8%	n=25 53.2%	100%	-
Age (average)	76.22	78.2	77.27	0.47
Tabagism	45%	24%	34.04%	0.35
Use of chest tube				
Hospital stay (average - days)	2.5	3.72	3.14	0.56
Chestu tube time (average - days)	12	13.28	12.68	0.55
Exsudate	13.63%	12%	12.70%	0.86
Transudate	86.36%	88%	87.30%	0.86
First drainage volume (average - mL)	1,120.45	1,114	1,183.82	0.96
Complications				
Infection	4.54%	4.20%	4.20%	0.92
Hard pain (recurent use of medication)	4.54%	17.02%	17.02%	0.019
Readmision after first drainage	27.27%	21.20%	21.20%	0.34

**Table 2:** Comparison of drain use and complications between smokers and non-smokers undergoing prolonged pleural drainage due to benign pleural effusion who were discharged with the drain. Variables were compared using the non-parametric Kruskal-Wallis test.

	Tabagists	Non-tabagists	p
Use of chest tube			
Hospital stay (average - days)	2.75	3.35	0.57
Chestu tube time (average - days)	12.43	12.8	0.87
Exsudate	18.75%	9.60%	0.41
Transudate	81.25%	90.40%	0.41
First drainage volume (average - mL)	1,131.25	1,180.64	0.77
Complications			
Infection	0%	6.45%	0.3
Hard pain (recurent use of medication)	12.50%	19.35%	0.52
Readmision after first drainage	43.75%	16.12%	0.04

**Figure 1:**
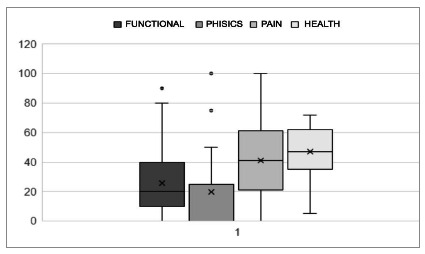
Graph representing the distribution and standard deviations of scores in the functional capacity, limitation due to physical aspects, pain, and general health domains, answered in the SF 36 questionnaire by patients undergoing pleural drainage for benign pleural effusion. Raw scale, no unit values.

**Figure 2:**
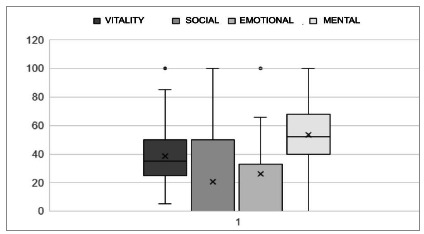
Graph representing the distribution and standard deviations of scores in the vitality, social aspects, limitation due to emotional aspects, and mental health domains, answered in the SF 36 questionnaire by patients undergoing pleural drainage for benign pleural effusion. Raw scale, no unit values.

The results of the quality-of-life analysis are presented in Figures 1 and 2.

## DISCUSSION

Even with all the evolution in medicine, the management of pleural effusions remains in evidence, perhaps due to its high prevalence, especially among elderly patients. Malignant pleural diseases have been studied more than benign ones. Brazilian and international studies show the outpatient care for patients with metastatic pleural effusions and the performance of outpatient pleurodesis, with good results and low infection rates^19^ ^,^ ^20^, in addition to the already established outpatient care for patients with pneumothorax^4^ ^,^ ^5^. Such evidence, associated with the fact that patients remained hospitalized for long periods only due to a pleural drain, led us to believe in good results with the outpatient use. In this prospective analysis, we observed patients with advanced ages, up to 95 years. This is because our service is specialized in the care of elderly patients. 

A limitation of the study was the sample of patients, which was initially calculated according to the healthcare provider’s population. The number of patients evaluated was imprecise in relation to the calculated sample, portraying the pragmatic nature of the study. Studies with more accurate sample size calculation and a larger number of patients will provide evidence of greater certainty. 

We chose to use the WC and not the IPC for this study for two reasons. The first is that the patients underwent drainage mainly to relieve symptoms, on an urgent basis. This routine does not fit the use of IPC. The second is that the cost of the IPC is higher, rendering it unfeasible on a large scale in group medicine. 

The short hospital stay time after drainage and the small percentage of readmissions due to pleural effusion show the effectiveness of the treatment. Patients feel more comfortable after drainage, which leads to faster compensation of comorbidities and shorter hospital stay. In addition to the cost reduction due to the shorter hospital stay, we also emphasize the lower probability of developing delirium and infection by multi-resistant microorganisms^21^. The 4.25% infection rate is comparable to that found in other studies, both for WC and for IPC^15^ ^,^ ^20^ ^,^ ^22^. 

Another variable analyzed was smoking, since 34.04% of the patients were active or former smokers. According to INCA data from 2013, the prevalence of smoking in the adult population is 14.7%^23^. Our data showed a much higher percentage of smokers, perhaps because it is a selected population, with chronic diseases, in which smoking plays an important role. Despite this, there was no statistically significant difference between smokers and non-smokers regarding complications, length of stay, and drainage time (Tables 1 and 2). 

As for pain, we chose to evaluate the use of analgesics as this is a continuous parameter. The assessment using the visual analogue scale, as some studies describe^15^ ^,^ ^16^ is performed at the time of return and, until then, the pain may have been significantly reduced. Thus, we observed that 65.95% of patients used analgesics occasionally and 23.40% did not use analgesics. 

Quality of life, analyzed according to the parameters of the SF 36 questionnaire - translated and validated for patients in Brazil, had low mean values, especially when compared with data from other studies^15^ ^,^ ^16^. In the analysis of each descriptor, the mean was higher only in the limitation by physical aspects. In the other descriptors, the results were similar, but smaller^16^. There are biases in the comparison, mainly because the population studied by these authors consisted of patients with neoplastic pleural effusions, and not composed exclusively of elderly people, as in the present study. Factors that may have contributed to the low values in quality of life are advanced age, which leads to great functional, social, and health limitations, in addition to clinical comorbidities, present in 100% of patients. We did not carry out pre-drainage quality of life assessment, as the patients were in a medical emergency situation, but we believe that all had an improvement in overall quality of life. As previously described, all patients reported improvement in their respiratory status after drainage.

Spontaneous pleurodesis is described in cases of prolonged use of pleural drains^9^ ^,^ ^16^. We did not objectively evaluate this in the present study, but the low rehospitalization rate related to pleural effusion (4.25%) suggests that it can occur with the use of WC as well.

The cost of using WC for benign effusions can also be assessed. With an average hospital stay of 3.14 days after the initial drainage and an average drainage time of 12.63 days, there is a reduction of 9.49 days of hospitalization, on average. Other studies have already shown better cost-effectiveness with the use of IPC for malignant effusions when compared with the usual care for the inpatient^8^ ^,^ ^22^. Thus, we believe that for the use of WC in benign effusions (simpler and cheaper than IPC) the analysis applies in a similar way.

Despite the small number of patients included, we believe that the results presented reflect the routine in the management of pleural effusions in elderly patients. A possible bias in the study is the lack of a control group, which could be obtained from hospitalized patients, but the assessment of quality of life in such individuals would possibly be altered by hospitalization. Furthermore, selection bias could occur, as more severe patients would be hospitalized. A point to be highlighted is that the entire process of placement and handling of the WC was carried out by a team of thoracic surgeons trained and dedicated to the service. This fact minimizes complications and standardizes conduct and procedures, highlighting the importance of the work of specialists for this type of patient.

## CONCLUSION

Early discharge and the use of outpatient Wayne pleural catheters (pigtail) proved to be feasible, safe, and with a low rate of associated infections. The quality-of-life analysis showed lower results than those of similar studies, but the populations studied were different. It is a viable option for selected patients.
